# Coagulopathy in elderly patients with coronavirus disease 2019

**DOI:** 10.1002/agm2.12133

**Published:** 2020-12-29

**Authors:** Xueting Yuan, Xunliang Tong, Yan Wang, He Wang, Liuming Wang, Xiaomao Xu

**Affiliations:** ^1^ The Key Laboratory of Geriatrics, Beijing Institute of Geriatrics, Beijing Hospital, National Center of Gerontology, National Health Commission; Institute of Geriatric Medicine, Chinese Academy of Medical Sciences P. R. China; ^2^ Department of Pulmonary and Critical Care Medicine Beijing Hospital, National Center of Gerontology; Institute of Geriatric Medicine, Chinese Academy of Medical Sciences P. R. China; ^3^ Tongji Hospital, Tongji Medical College, Huazhong University of Science and Technology Hubei China

**Keywords:** aged, blood coagulation disorders, COVID‐19, inflammation

## Abstract

**Background:**

Since the outbreak of coronavirus disease 2019 (COVID‐19), clinical features have been analyzed in detail. However, coagulopathy in elderly COVID‐19 patients has been scarcely reported.

**Methods:**

Coagulation parameters of 189 patients with COVID‐19 in Tongji hospital were retrospectively analyzed among age groups.

**Results:**

Patients were divided into 2 groups: older group (≥65 years, n = 87) and younger group (<65 years, n = 102). The proportion of patients with elevated fibrinogen (79.0% vs 59.6%, *p* = .005) and D‐dimer (78.0% vs 55.2%, *p* = .001) shows the significant difference between the groups. The elderly patients revealed significantly longer prothrombin time (14.0 [13.4–14.4]s vs 13.6 [13.2–14.1]s, *p* = .026), higher D‐dimer (1.00 [0.5–1.9] μg/mL vs 0.6 [0.3–1.6] μg/mL, *p* = .013) and fibrinogen (5.2 [4.1–6.2] g/L vs 4.4 [3.4–5.7] g/L, *p* = .004) levels, compared to the younger group. A positive correlation was observed between the coagulation parameters and inflammatory markers including high‐sensitivity C‐reactive protein and interleukin‐6 (*p* < .05).

**Conclusions:**

The hypercoagulable state is more common in elderly COVID‐19 patients, and coagulopathy is associated with excessive systemic inflammation.

## Graphical abstract

In this study, we summarized the characteristics of coagulopathy in different age groups with SARS‐CoV‐2 infection, and investigated the relations between coagulopathy and systemic inflammation in COVID‐19 patients.

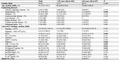

## 1 INTRODUCTION

In December 2019, an outbreak caused by coronavirus disease 2019 (COVID‐19) occurred in Wuhan, Hubei Province, China. The causative agent of COVID‐19 is a novel β‐coronaviruses named SARS‐CoV‐2,^1^, ^2^ which shares homology with two bat‐derived SARS‐like coronaviruses and distances from severe acute respiratory syndrome coronavirus (SARS‐CoV, around 79%) and Middle East respiratory syndrome coronavirus (MERS‐CoV, around 50%).^3^, ^4^ The effect of SARS‐CoV‐2 infection on coagulation is considered similar to pneumonia induced by SARS‐CoV^5^, ^6^ and MERS‐CoV.^7^, ^8^ COVID‐19‐associated coagulopathy is common in patients, causing high rates of thrombotic complications that increase the mortality.^9^, ^10^ Previous study shows that older individuals aged above 50 years had a higher risk of thrombosis.^11^ Platelets and other factors independent of the coagulation cascade, play a more significant role in age‐related thrombosis.^12^ However, complete coagulation parameters of COVID‐19 patients in the elderly were not fully reported. Markedly elevated levels of D‐dimer are the hallmark laboratory findings and correlate with severity of illness and risk of thrombosis.^10^ Appropriate venous thromboembolism (VTE) prophylaxis is very important for all patients with COVID‐19. In this study, we aimed to summarize the characteristics of coagulopathy in different age groups with SARS‐CoV‐2 infection, and to investigate the relations between coagulopathy and systemic inflammation in COVID‐19 patients.

## 2 MATERIALS AND METHODS

### 2.1 Study design

A 189 patients with severe COVID‐19 admitted to the Sino‐French New City Branch of Tongji Hospital in Wuhan from January 28 to March 8, 2020, were retrospectively enrolled. A confirmed case of COVID‐19 was defined as a positive result on real‐time reverse transcription–polymerase chain reaction (RT‐PCR) assay of nasal and pharyngeal swab specimens. The severity of the disease was assessed according to the Seventh Version of the Novel Coronavirus Pneumonia Diagnosis and Treatment Guidance from the National Health Commission of China. Pneumonia patients only infected with bacteria, fungi, and other atypical pathogens were excluded. Coagulation parameters were compared among age groups (≥65 years vs <65 years), and the correlations between coagulation and inflammation were analyzed. The study was approved by the Ethics Committee of Beijing Hospital(2020BJYYEC‐046‐01).

### 2.2 Data collection

The clinical data, including demographics information; underlying diseases; laboratory results and clinical outcomes were extracted from electronic medical records. Coagulation tests included prothrombin time (PT), activated partial thromboplastin time (APTT), fibrinogen (FIB), platelet count (PLT) and D‐dimer. High sensitivity C‐reactive protein (hsCRP) and interleukin‐6 (IL‐6) as inflammatory markers were also detected. The data above were recorded in the first 24 hours after diagnosis. The end point was written of discharging from hospital or death. All the data were reviewed by experienced physicians separately and checked by two physicians independently.

### 2.3 Statistical analysis

All measurements were expressed as median (interquartile range [IQR]) or number (%). Continuous variables were compared using the Mann‐Whitney U test. Categorical variables were compared using the Chi‐squared test. Correlations between variables were analyzed using the Spearman's rank correlation. A *p*‐value < .05 was considered statistically significant. Data was analyzed using SPSS software (version 25.0).

## 3 RESULTS

A total of 189 patients with severe COVID‐19 were enrolled in this study. The median age was 59.2 years (range, 24–91 years) and 95 (50.3%) were male. 91(46.0%) patients had chronic underlying diseases, including chronic respiratory disease (6.3%), hypertension (41.5%), diabetes (34.8%), cardiovascular diseases (5.3%), cerebrovascular diseases (2.1%), tumors (3.2%), liver diseases (0.5%), and kidney diseases (3.2%). All patients were treated according to the pneumonia diagnosis protocol for novel coronavirus infection (trial version 7).^2^ By the end of March 21, 2020, 178 patients (94.2%) were discharged and 11 (5.8%) died, including 6 cases in the older group and 5 cases in the younger group.

### 3.1 Comparison of coagulopathy among age groups

Patients were divided into 2 groups: older group (≥65 years, n = 87) and younger group (<65 years, n = 102). The proportion of patients with elevated FIB (79.0% vs 59.6%, *p* = .005) and D‐dimer (78.0% vs 55.2%, *p* = .001) shows the significant difference between the groups. The elderly patients revealed significantly longer PT (14.0 [13.4–14.4]s vs 13.6 [13.2–14.1]s, *p* = .026), higher D‐dimer (1.00 [0.5–1.9]μg/mL vs 0.6 [0.3–1.6]μg/mL, *p* = .013) and FIB (5.2 [4.1–6.2]g/L vs 4.4 [3.4–5.7]g/L, *p* = .004) levels, compared to the younger group. Further clinical details are summarized in Table 1.

**Table 1:** Coagulation parameters of different age groups

	Total	<65 years old (n = 102)	≥65 years old (n = 87)	*p*
Gender, Male	95/94	55/47	40/47	.308
Age, median (IQR), years	63.0 (49.0–69.0)	49.5 (39.8–59.0)	70.0 (67.0–86.0)	–
Underlying diseases				
Chronic respiratory disease (%)	6.3 (12/189)	5.9 (6/102)	6.9 (6/87)	.776
Hypertension (%)	41.5 (76/183)	27.5 (28/102)	55.2 (48/87)	**.000**
Diabetes (%)	34.8 (31/189)	12.7 (13/102)	20.7 (18/87)	.142
Cardiovascular disease (%)	5.3 (10/189)	2.9 (3/102)	8.0 (7/87)	.118
Liver diseases (%)	0.5 (1/189)	1.0 (1/102)	0	–
Kidney diseases (%)	3.2 (6/189)	1.0 (1/102)	5.7 (5/87)	.062
Cerebrovascular disease (%)	2.1 (4/189)	2.0 (2/102)	2.3 (2/87）	.872
Malignant tumor (%)	3.2 (6/189)	1.0 (1/102)	5.7 (5/87)	.062
Laboratory findings, median (IQR)				
Platelets, ×10^9^/L	225.0 (156.3–304.3)	217.0 (146.0–306.0)	231.0 (182.0–297.0)	.362
Platelets < 100 (×10^9^/L)(%)	6.5 (12.0/186.0)	7.1 (7/99)	5.7 (5/87)	.714
PT(s)	13.8 (13.3–14.3)	13.6 (13.2–14.1)	14.0 (13.4–14.4)	**.026**
PT > 14.5s(%)	15.5 (29/187)	12.0 (12/100)	19.5 (17/87)	.155
APTT(s)	40.1 (36.7–44.2)	39.7 (37.2–43.2)	41.0 (36.1–45.1)	.791
APTT > 42s(%)	35.5 (65/183)	33.0 (33/100)	39.8 (33/83)	.343
FIB (g/L)	4.8 (3.8–5.9)	4.4 (3.4–5.7)	5.2 (4.1–6.2)	**.004**
FIB > 4.0 g/L (%)	68.3 (123/180)	59.6 (59/99)	79.0 (64/81)	**.005**
D–Dimer, μg/mL	0.7 (0.4–1.8)	0.6 (0.3–1.6)	1.0 (0.5–1.9)	**.013**
D–Dimer ≥ 0.5 μg/mL (%)	66.3 (118/178)	55.2 (53/96)	78.0 (64/82)	**.001**
hsCRP(mg/L)	24.0 (4.1–51.2)	16.1 (3.3–44.0)	25.5 (5.6–70.3)	.100
hsCRP ≥ 1mg/L (%)	92.9 (144/155)	90.8 (79/87)	95.6 (65/68)	.250
IL–6 (pg/mL)	6.6 (2.5–21.4)	6.4 (1.8–19.2)	9.4 (4.1–28.3)	.09
IL–6 ≥ 7 pg/mL (%)	48.9 (44/90)	46.7 (21/45）	55.6 (25/45)	.399
Death (No. (%))	5.8 (11/189)	4.9 (5/102)	6.9 (6/87)	.559

### 3.2 The grade of disseminated intravascular coagulation

According to the Consensus of Chinese experts on the diagnosis of disseminated intravascular coagulation (version 2017),^13^ two patients matched the grade of DIC (≥7 points). One of the patients complicated with diffuse large B‐cell lymphoma and the other patient had diabetes. None of the survivors matched the diagnosis of DIC in this study.

### 3.3 Correlation between coagulation and inflammation

**Figure 1:**
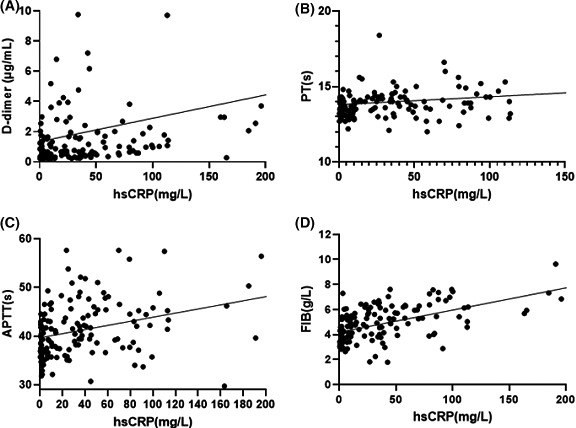
Correlation between hsCRP and Coagulation parameters

**Figure 2:**
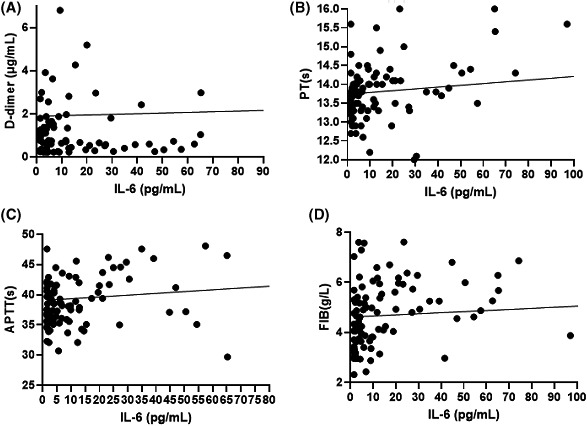
Correlation between IL‐6 and Coagulation parameters

In enrolled COVID‐19 patients, 144 (92.9%) had elevated hsCRP levels, and 44 (48.9%) had elevated IL‐6 levels. hsCRP levels were significantly correlated with PT(ρ = 0.293, *p* < .001), APTT(ρ = 0.412, *p* < .001), FIB(ρ = 0.598, *p* < .001) and D‐dimer(ρ = 0.439, *p* < .001) concentrations (Figure 1); IL‐6 levels were positively correlated with PT(ρ = 0.317, *p* < .001), APTT(ρ = 0.380, *p* < .001), FIB(ρ = 0.590, *p* < 001) and D‐dimer (ρ = 0.419, *p* < .001) concentrations, which showed with statistical differences (Figure 2) (ρ: correlation coefficient).

## 4 DISCUSSION

Coagulation system plays a crucial role in the innate immunity.^14^ Multiple inhibitory pathways regulate the coagulation process, such as tissue factor pathway inhibitor (TFPI), antithrombin (AT), and protein C system.^15^ The imbalance between coagulation and anticoagulation systems is responsible for abnormalities. Current evidence points to an extensive cross‐talk between coagulation and inflammation systems.^16^ COVID‐19 infection has a heterogenous disease course, majority of patients only have mild symptoms, while some severe patients have immunologic complications, resulting in cytokine storm.^17^ Inflammatory factors such as IL‐1 and IL‐6 promote a hypercoagulable state through excess thrombin generation and fibrinolysis shutdown.^18^ Previous studies have asserted that advanced age is a risk factor in the development of VTE and that the incidence of VTE increases significantly with increasing age.^19^, ^20^ COVID‐19 patients with coagulation dysfunction have a higher risk of death.^21^ Therefore, coagulopathy in elderly COVID‐19 patients need more clinical concern.

Klok FA et al reported a VTE rate of 27% in severe COVID‐19 patients, and pulmonary embolism (PE) was the most frequent thrombotic complication.^22^ Elderly patients always have reduced physical mobility and high hospitalization rates for illnesses that contribute to the development of VTE.^20^ In our study, we found higher D‐dimer and FIB levels in the older group, which indicated that elderly COVID‐19 patients tend to be in a hypercoagulable state and have higher risks of thromboembolism. Besides, longer PT was also observed in the older group compared to the younger group but both groups were within the normal reference range and did not belong to the pathological state of the disease, which may be considered age‐dependent. Due to limited conditions, we failed to evaluate the incidence of patients with VTE by computed tomography pulmonary angiography (CTPA) and ultrasound examination. Given the potential risk factors, such as infection, bed rest, and respiratory failure,^23^ we took preventive measures for patients with high risks of VTE.

Elevated D‐dimer levels on presentation with COVID‐19 are associated with severe disease.^23^, ^24^, ^25^ In our study, 66% of patients exhibited dramatically elevated levels of D‐dimer and 82% of the death had D‐dimer ≥ 1 μg/mL on admission, confirming that D‐dimer may be a helpful predictor for prognosis. The elevation of D‐dimer is affected by many factors, such as tumor, pulmonary embolism, inflammatory factors, drugs and so on. The clinical characteristics of hypercoagulable tendency in older patients were more significant in this cohort, which deserved more attention at the early stage. And independent risk factors affecting D‐dimer need to be further explored by more prospective and multicenter studies. DIC appeared in most of the deaths,^23^ and two patients matched the diagnosis of DIC in our study, which is the result of coagulation activation and secondary hyperfibrinolysis condition at the late stages of COVID‐19. Several studies showed that prophylactic anticoagulant can benefit severe COVID‐19 patients,^26^ whereas the treatment duration and intensity warrant further study.

Current evidence indicates that COVID‐19‐associated coagulopathy is the result of cytokine storms triggered by SARS‐CoV‐2.^27^, ^28^, ^29^ Previous study confirmed that intense inflammation may aggravate the damage to organs and excessive consumption of clotting factors may increase the risk of thromboembolism.^30^ In this study, we found hsCRP and IL‐6 were significantly correlated with abnormal coagulation parameters. Continuous monitoring of coagulation can predict the time of inflammatory storms to guide the clinical use of anti‐inflammatory drugs. The specific mechanism of coagulopathy induced by SARS‐CoV‐2 needs further investigation.

Only 189 patients were included in our cohort due to the time and condition constraints. Nonetheless, our study has shown that the hypercoagulable state is more common in elderly COVID‐19 patients, with the longer PT, higher D‐dimer and FIB levels. HsCRP and IL‐6 levels had significant correlations with coagulation parameters, which confirmed that coagulopathy is associated with excessive systemic inflammation. The monitoring of the coagulation function may be helpful to evaluate early prognosis in the elderly.

## CONFLICT OF INTEREST

The authors report no conflicts of interest related to the submitted work.

## AUTHORS' CONTRIBUTIONS

Designed research: Xiaomao Xu, Xueting Yuan and Xunliang Tong; performed research: Xueting Yuan and Xunliang Tong; collected data: Xunliang Tong, Yan Wang, He Wang, Liuming Wang and Xueting Yuan; analyzed data: Xueting Yuan and Xunliang Tong; wrote the paper: Xueting Yuan, revised the paper: Xunliang Tong, Yan Wang and Xiaomao Xu.

## Data Availability

All the data from electronic medical records in Tongji Hospital were reviewed by experienced physicians separately and checked by 2 physicians independently.
